# Evaluation of oral mucosal epithelium in diabetic male patients by exfoliative cytology method

**DOI:** 10.1186/2251-6581-13-77

**Published:** 2014-09-10

**Authors:** Safoura Seifi, Farideh Feizi, Zoleikhah Moazzezi, Mohammad Mehdizadeh, Babak Zamani

**Affiliations:** Department of Oral and Maxillofacial Pathology, Babol Dental University, Babol, Iran; Department of Histology, Babol Medical University, Babol, Iran; Department of endocrinology, Babol Medical University, Babol, Iran; Department of Oral and Maxillofacial Surgery, Babol Dental University, Babol, Iran; Dentist, Babol, Iran

**Keywords:** Diabetes mellitus, Cytomorphomerry, Oral exfoliative cytology

## Abstract

**Background:**

The goal of present study is to examine comparatively the epithelium of oral mucosa in persons with diabetes types I and II and the healthy persons by cytomorphometric method.

**Methods:**

Cytology smear was prepared from oral mucosa epithelium in 24 persons with diabetes and 30 healthy persons were stained by papanicolao method. Its before, from patients were requested acquiring written. The quantitative cytomorphometric characteristics were measured in each group by motic plus 2 software. Also, the qualitative evaluation of cytology slides are performed in three groups.

**Results:**

There were decrease in the nuclear and cytoplasmic size, (P < 0.001) and increase in the ratio of nuclear to cytoplasm size in buccal mucosal areas (P = 0.001) and tongue (P = 0.011) of diabetic persons compared to the healthy persons. There was no significantly statistical difference in diabetic persons types I and II in buccal mucosal area (P = 0.15) and tongue (P = 0.86) in quantitative characteristics of cytomoerphometry. In nuclear and cytoplasm size, there was a significant statistical difference in persons with diabetes type I and persons of control I and persons with diabetes type II and persons of control II in buccal mucosa and the tongue (P < 0.001). Bilobed or multi-lobed nuleous, karyorrhexis and vacuolization of cytoplasm were more in diabetic persons compared to the healthy ones (P < 0.001).

**Conclusion:**

Diabetes are effective in creating some quantitative and qualitative cytometric alterations in the oral mucosa but the type of diabetes doesn’t seem to be effective on these changes.

## Introduction

Diabetes mellitus is a complex metabolic disease which is followed by disorders in the metabolism of carbohydrate, lipid and protein [1]. It’s incidence is increasing in developing countries [2] and mortality is resulted from this disease in the performance of vascular system and default in the activity of kidneys [3]. It includes diabetes types I and II, gestational diabetes and diabetes type 1.5 or double type. Diabetes type II is the most prevalent kind of diabetes but diabetes type I has a slight incidence. Diabetes type 1.5 occurred in the adults affected by diabetes who have the characteristics of diabetes types 1 and 2 [4]. Diabetes in the performance of oral mucosa due to the substitution and change in the salivary amount and change in the kind of nutrition and decrease in the activity of immune system. Oral problems of persons suffering from diabetes include oral dryness, candidiasis, increase in teeth decay, inflammation of gingiva, periodontitis, peri-apical abscess [5, 6]. There are several methods to evaluate the oral mucosa of persons with diabetes which one of the most prevalent of these methods is the application of biopsy as incisional or exisional but using of biopsy is usually not applied due to being aggressive and creating cycological problems for the patient [7]. It seems that the best method with low cost and less aggressive characteristic and lack of damage to oral tissues of the patient, is using of cytology exfoliative or brush cytology which was initially applied for rapid diagnosis of precancerous lesions but today, it is used for diagnosis and evaluation of the quantitative and qualitative changes in epithelial cells of the oral mucosa is suspected [8, 9]. Cytology is considered to be as a supplementary method which is fast, safe and without need anesthesia. Papanicolau is the easiest and most common cytology technique for smear staining [10]. Cytometry is a technique for characterization and measurement of cells and cellular specification such as nucleous and cytoplasm size, nuclear-cytplasmic ratio, aneuploidy/diploidy analysis of nucleus with using images for microscopic slides coupled with attached camera system which are measured using special software [11].

In total; up to date, a few studies examined changes in the oral mucosa of diabetic patients and reported about the replacement change in epithelial cells of the oral mucosa by cytology method [1, 3] and the comparison of cytomorphometric changes of the oral mucosa of persons with diabetes types I and II was reported just in one study [12]. Therefore, the goal of the present study is to examine the quantitative and qualitative characteristics of the buccal mucosa and tongue epithelium by exfoliative cytology in patients with diabete types I and II and compare them to each other and to the healthy persons.

## Methods

The study was approved by the Ethics Committee of Babol university. In this case–control study, 24 diabetic persons (9 persons with diabetes type I and 15 persons with diabetes type II) were selected from endocrinologist office in babol. The age, gender, disease duration and type of diabetes, their medical history were recorded in the pre-prepared tables. Also, 30 healthy persons who had no factors for diabetes development and their blood sugar was less than 120 mg/dl, are considered as control group. The healthy persons were selected from boy students, dormitory and the staff of dentistry department. smokers, alcoholic persons, persons with anemia and malignancy and any kind of systemic problem, oral lesions and gingivitis and periodontal disease in each group were excluded regarding to the possibility of their affection in the cellular form and morphology.

The control group in the healthy persons were homoscedasticized with the case group (diabetic persons) in terms of age and gender. In order to increase the precision of the study, two control groups for diabetes types I and II were separately considered.

It should be mentioned that determination of the type of diabetes was performed by endocrinologist based on the criteria of Japan’s diabetes association in diagnosis and classification of diabetes [13], so that persons with a 8–10 years record for diabetes affliction and under control of endocrinologist and at least having one of the following characteristics, were included in the study.

1. serum glucose level randomly as 200 mg/dl or ≥11.1 mm.
2. Initial serum glucose level as 126 (FBS) mg/dl or ≥ 7.omm.
3. Two- hour glucose of plasma as 2 hp 200 mg/dl or ≥ 11.1 mm.

Before preparation of smear, the goal of the study and its advantages were explained for the persons and after acquiring written consent, their mouths were washed by water and a piece of gauze was slowly drawn on the desired regions. The examination and confidence from the absence of food particles was performed by two single-use cytobrushes (cytobrush, pad Tan Teb, Iran) and the smear was prepared individually and separately from each of oral regions and right-side tongue.

In each site, cytobrush was rotated 10–17 times and bleeding was not produced in any of the samples and then, each brush related to its own anatomical area was extended on the dry and clean glassy slide which wase pre-labelled and numbered and immediately, the existing cells on the surface of slide were fixed by pathofix spray containing ethanol 95% (Tehran-Iran pad tan Teb) by a distance of 25 cm by maximum 2 times spray pressure and the code related to every patient was written on each slide and in order to prevent the possible error, all samples were, transtferred to pathology laboratory of dentistry department and during 1–3 days later, they were stained by papanicolao color. Cytology samples were placed into different degrees of alcohol (concentrated to diluted) (90 degrees, 70 degrees, 50 degrees), respectively. And then, they were placed into the distilled water and then, they were placed into papanicolao stain for 5–10 minutes.

Then they were placed into the distilled water and alcoholic acid 5% and then, they were exposed on the distilled water and lithium carbonate and again, they were washed into the distilled water and 50, 70 and 90 degrees alcohols and then, they were placed into orange solution alcohol 95 degrees for one minute, and then into papanicolao staining for 2 minutes, and again, they were placed into the alcohol 95, absolute alcohol and xylene for five minutes and were covered by lamel. To evaluate cytomorphometry, the computer connected to camera and photoshop software and the analysis system type Motic Image plus 2 (micro- optic industrial group co LTD1) was used.

Imaging from slides was performed with 40-fold magnification by light microscope. In each slide, an average of 50 cells with strong staining and the determined cellular limits were selected. If placing cells on top of one another was observed and their membranes were not obvious, they were excluded the study. In order to prevent the errors of measurement and enumeration of cellular samples again, the movement of microscope is performed from left to right, then to up and down. Afrer that, the determination of the average sizes of the nucleous and cytoplasm of each cell (in *μm*^2^) and the ratio of nuclear to cytoplasm size were performed and the results were reported in Mean ± SD.

### Qualitative evaluation of cytomorphometry

In each slide, cytology by a number of 50 cells in 5 microscopic fields with the magnification of 40- fold were examined in terms of the characteristics of the nucleous (the presence of bi-lobed or multi-lobed nucleous, karyorrhexis, vacuolization of cytoplasm, the presence of inflammation and candida in buccal mucosal areas and lateral tongue (right side) in persons with diabetes types I and II and the healthy persons and the mean results was expressed in percent.

### Statistical analyses

The results were introduced into SPSS (17) and the comparison between three groups and the statistical analyses was performed and p-value less than 0.05 (P < 0.05) was considered as significant.

In order to compare the size of nucleous, cytoplasm and the ratio of the nuclear to cytoplasmic size in two regions of tongue and buccal mucosa in two groups of diabetic persons and the healthy ones, T-test was used and for comparison of the above- mentioned variables in four groups of diabetes I and diabetes II and the healthy persons, Kruskall–wallis and Mann-witney tests were used. For comparison of the qualitative characteristics of cytomorphometry in four groups of persons with diabetes I, persons with diabetes II, and the healthy persons (control I, control II ), Chi-square analysis was used.

## Results

In terms of age, there was no significant difference between persons with diabetes type I and those of control I and persons with diabetes type II and those of control II (P = 0.1 ) (Table 1).

**Table 1:** **Demographic data** (**age and gender**) **of persons with diabetes types I and II. and the related control group**

Demographic characteristics	Diabetes type I	Control I	Diabetes type II	Control II
**Age**	24.33 ± 2.5	25 ± 2.93	34.07 ± 8.23	33.67 ± 3.44

Demographic data (age) of patients with diabetes types I and II. and the healthy persons were summarized in Table 1.

There was a statistically significant difference in terms of nuclear and cytoplacsmic size, (P < 0.001), the ratio of nuclear to cytoplasmic size in persons with diabetes and the healthy persons in buccal mucosal area (P = 0.001) and tongue region (P = 0.011) (Table 2).

There was a statistically significant difference in terms of nuclear and cytoplasm size (P < 0.001) and the ratio of nuclear to cytoplasmic size (P = 0.02) in persons with diabetes type I and control I group in buccal mucosal area.

**Table 2:** **Mean size of the nucleous**, **cytoplasm and the ratio of nuclear to cytoplasmic size in diabetic persons and the healthy ones in buccal mucosa and tongue regions**

Healthy person patient	Number	Location of smear preparation	Nuclear size ***μm*** ^2^	Size of cytoplasm ***μm*** ^2^	Ratio of nuclear to cytoplasm size (N/C)
**Diabetic person I**	9	**Buccal mucosa**	1752.92 ± 422.5***	58149.015 ± 20367.77***	0.049 ± 0.023***
**All diabetic patients**	24		1752.92 ± 422.85***	58149.015 ± 20367.77***	0.049 ± 0.023***
**Healthy person**	30		3124.37 ± 744.97	115669.16 ± 45630.3	0.049 ± 0.004
**Diabetic person II**	15	**Tongue**	1746.29 ± 577.95***	56236.34_±_16963.85***	0.053_±_0.032**
**All diabetic patients**	24		1746.29 ± 577.95***	56236 ± 16963.85***	0.053 ± 0.032**
**Healthy person**	30		4263.23 ± 1336.33	178922.92 ± 50093.05	0.0359 ± 0.002

**P=0/001.

***P=0.001.

*μm*: *μ*icrometer.

- In terms of nuclear and cytoplasm size (P < 0.001) and the ratio of unclear to cytoplasm ratio (P = 0.016), there was statistically significant difference between persons with diabetes type II and those of control II group in buccal mucosal area (Table 3).

**Table 3:** **Mean size of nucleous**, **cytoplasm and the ratio of nuclear to cytoplasm size in persons with diabetes types I and II and control groups** (**I and II**) **in buccal mucosal area**

Healthy person/ patient	Size of nucleous ***μm*** ^2^	Size of cytoplasm ***μm*** ^2^	Ratio of nuclear to cytoplasm size (N/ C)
Diabetic I	1831.37 ± 340.82***	61604.32 ± 22264.***	0.06 ± 0.03***
Control I	3048.55 ± 666.34	126220.65 ± 49069.6	0.0307 ± 0.00047
Diabetic II	1705.86 ± 470.15	56075.83 ± 19.646***	0.042 ± 0.013*
Control II	1384.1 ± 877.74***	99391.93 ± 35865.48***	0.0306 ± 0.00052

*P=0.05.

***P=0.001.

There was a statistically significant difference in terms of the size of nucleous and cytoplasm (P < 0.001) in tongue region between persons with diabetes type I and those of control I and persons with diabetes type II and those of control II.

- There was no statistically significant difference in terms of the ratio of nuclear to cytoplasm in tongue region in persons with diabetes type I and those of control I (P = 0.21) and persons with diabetes type II and those of control II (P = 0.39) (Table 4).

**Table 4:** **Mean size of nucleous and cytoplasm, the ratio of nuclear to cytoplasm in persons with diabetes types I and II and control groups of I and II in tongue area**

Healthy person/ patient	Size of nucleous ***μm*** ^2^	Size of cytoplasm ***μm*** ^2^	Ratio of nuclear to cytoplasm size (N/ C)
Diabetic I	1743.8 ± 408.82***	58297.84 ± 16412.68***	0.05 ± 0.024***
Control I	4219.78 ± 1421.63	171014.23 ± 48186.09	0.0352 ± 0.00257
Diabetic II	1747.75 ± 673.23***	54999.43 ± 17733.20***	0.05 ± 0.037*
Control II	4328.4_±_1255.72	190785.94_±_52643.43***	0.0347_±_0.00049

*P=0.05.

***P=0.001.

### Qualitative evaluation of cytology smears

There was a statistically significant difference in terms of candidate (P = 0.001) and inflammation (P < 0.001) between diabetic persons and the healthy ones in buccal mucosal region but this difference was not significant in the tongue region (P = 1).

In comparison between persons diabetes I and diabetes II, there was no statistically significant difference in buccal mucosa areas in terms of inflammation (P = 0.32) and candidate (P = 1). This difference was not statistically significant in tongue area (P = 1).

**Figure 1:**
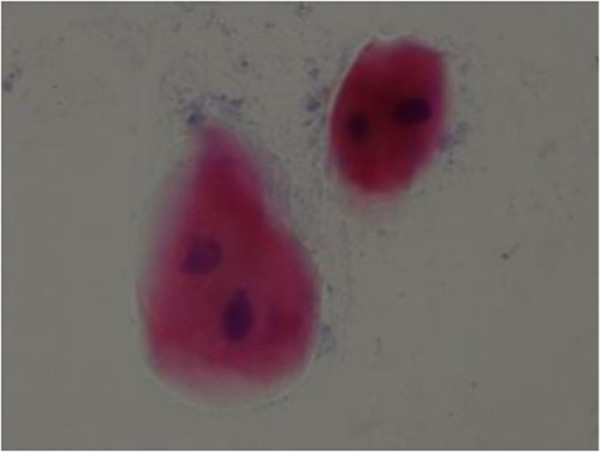
**Smear cytology from buccal mucosal region in diabetes type II.** Bi-lobed nucleous, papanicolao staining (×100).

**Figure 2:**
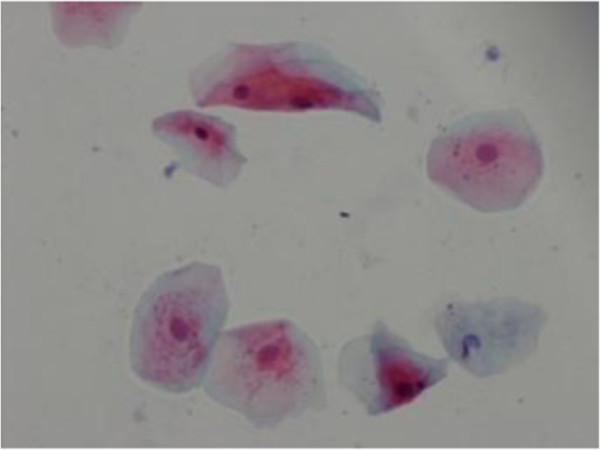
**Smear cytology from buccal mucosa of the diabetes type I.** karyorrhexis, cytoplasm vacuolization, Bi- lobed nucleous with papanicolao staining (×40).

There was statistically significant difference in terms of bi-lobed nucleous or multi-lobed nucleous, karyorrhexis and cytoplasm vacuolization in diabetic persons and the healthy ones in tongue and buccal mucosal areas (Table 5) (Figures 1 and 2).

**Table 5:** **The comparison between the situation of nucleous** (**bi**-**lobed or multi**-**lobed**, **karyorrhexis**) **and vacuolization of the cytoplasm in diabetic persons and the healthy ones in tongue and buccal mucosal region**

Diabetic/ Healthy	Site of smear preparation	Bi-lobed or multi- lobed nucleous	Karryorhexis	Cytoplasm vacuolization
Buccal mucosa	Diabetic	76%***	92%***	95.6%***
	Healthy	21.4%***	31.6***	26.9%***
Tongue	Diabetic	76%***	84%***	100%***
	Healthy	20%***	27.3%***	20% ***

***P=0.001.

## Discussion

Based on the results of the present study, there is a significant difference in nuclear and cytoplasm size, the ratio of nuclear to cytoplasm ratio in diabetic patients compared to the healthy persons. It seems that diabetes results increating quantitative cytomorphometric changes in the oral mucosa which from this perspective, the results of our study are similar to the results of studies by Jajarm [1], survarna [14], Shareef [3], Prasad [15], Alberti et. al. [9], but what changes this systemic diabetes disease creates in quantitative cytomorphometry characteristics of the oral mucosa, seems different in various studies.

In this study, the size of nucleous and cytoplasm were smaller in diabetic persons compared to the healthy ones but the ratio of nuclear to cytoplasm size was bigger. It is supposed that cell’s size in diabetic patients is smaller than that of the healthy persons.

On the other hand, epithelial cells of the oral mucosa are atrophied in diabetic persons and the size of nucleous and cytoplasm decrease, such that the formation of multiple wounds and oral infections indicate the atrophy of the oral mucosa in diabetic persons [1] and this athrophy is due to decrease in the volume and number of cells. The results of the study performed Calderia et.al. on the laboratory and diabetic rat represent the epithelial atrophy of the oral mucosa with cellular polymorphism and decrease in the number of the diagnostic cellular layers and decrease in the number of cellular organs [16].

Some researchers believed that because of ischemia and atherosclerosis resulted from diabetes, epithelial cells of the oral mucosa reach the oldness stage more rapidly and due to accumulation of incomplete proteins inside the core and decrease in cellular turnover, the nuclear size increases [17] but the production and intensity of atherosclerosis are different based on clinical stages of diabetes.

It seems that by increase in the damaging incentives and development of cell aging in diabetic patients because of decrease in the production of proteins, nucleic acids and cellular receptors, the number of cellular organelles and the nuclear and cytoplasm’s size decrease [18]. Just in the study of Abdulsamide et. al. decrease in the size of nucleous in diabetic patients is reported [12] which is similar to the above-mentioned study. The reason for difference between the results of the present study and those of other researchers in terms of the nuclear size, depends on the duration of affliction to diabetes and being controlled or uncontrolled of diabetes and in what stage of diabetes, smear cytology has been drawn from the oral mucosa.

In this study, the patients had a 8–10 years record for affliction to diabetes and decrease in the nuclear size represents the development of aging process in epithelial cells of the oral mucosa.

In diabetic patients, due to the produced hyperglycemia, disorder in repairing of the damaged tissues, disorder in secretion of saliva and xerostomia will be occurred.

The reason for xerostomia is because of systemic dehydratation, using of drugs and membranopathy of salivary ducts [3] and due to dehydration [2], cytoplasm size of epithelial cells will decrease. In this study, since decrease in the size of cell’s cytoplasm is more than decrease in the size of healthy cells’ cytoplasm’s because of dehydratation, consequently, the ratio of the nuclear to cytoplasm size in diabetic patients is more than that of the healthy persons. Abdolsamadi et. al. observed statistically significant difference in terms of the nuclear and cytoplasm size and the ratio of nuclear to cytoplasm in diabetes types I and II [12]. In our study , there was no statistically significant difference in the quantitative cytomorphometry characteristics in patients with diabetes types I and II.

It seems that the most important reason for the difference between the results of our study and theses of the research by Abdolsamadi et al. [12] is due to the measurement method. In our study, there was no statistically significant difference in the quantitative cytomorphometry characteristics in patients with diabetes types I and II.

It seems that the most important reason for the difference between the results of our study and thoses of the research by Abdolsamadi et al. [12] is due to the measurement method. In our study, motic plus 2 software and 40- fold (×40) microscopic magnification were used but they implemented counting point method and magnification of 100× in their study.

In relation to cytoplasm size in diabetic patients, contradictory results are obvious in different studies. Some of researchers reported increase in the size of cytoplasm [1], some others reported decrease in the size of cytoplasm [12, 14] and some reported lack of change in the size of cytoplasm in diabetic patients compared to the healthy persons [3, 9, 18]. In terms of decrease in the size of cytoplasm in diabetic patients, the above-mentioned study is similar to the studies of Tozoglu et al. [19] and Abdolsamadi et al. [12], but it is different from the results of studies performed by Jajarm [1], Shareef [3], Alberti et al. [9].

Some researchers regarded the increase in the ratio of the nuclear to cytoplasm’s size as the pre-cancerous and malignancy symptom [20] and some believe that there is a relationship between increase in the ratio of the nuclear to cytoplasm size and the cancer [21].

But some studies reported that because of the absence of the relationship between diabetes and cancer, it is better to use the ratio of cytoplasm to the nuclear size and they don’t explain about the lack of relationship between diabetes and cancer [3].

From the results of the present study, it seem that there is no relationship between diabetes and cancer, although the ratio of nuclear to cytoplasm increases in diabetic patients but the nuclear size in diabetic patients shows a decrease compared to that of the healthy persons. In malignancies, the increase in the nuclear size and the ratio of nuclear to cytoplasm size is usually parallel to each other [6]. Although in terms of increase in the ratio of the nuclear to cytoplasm size and on the other hand, decrease in the ratio of cytoplasm to the nucleous most studies are similar to the above- mentioned study but in our study, the increase in the ratio of the nuclear to cytoplasm is not resulted form decrease in the cytoplasm area. Just in the study of Abdolsamadi et.al, diabetes types I and II are compared to each other in terms of quantitative cytomorphometry chages in the oral mucosa [12] and this examination was not performed in any other study.

In the above-mentioned study, in comparison of the nuclear size, cytoplasm size and the ratio of the nuclear to the cytoplasm, inflammation and candida, there was not statistically significant difference between patients with diabetes types I and II. This finding confirms that changes in the size of nucleous and cytoplasm are resulted from hyperglycemia produced in diabetes and pathophysiology if the disease has no effect on production of these changes but in comparison of the quantitative cytomorphometric changes in patients with diabetes type I and the control I as well as the patients with diabetes II and the control II, there is an obvious statistically significant difference which indicates to the effect of diabetes types I and II on the quantitative cytomorphometry change of the oral mucosa.

Some researchers believe that the age and gender effective on the quantitative cytomorphometry characteristics of the mouth [22] and other studies reject these results [15].

In this study, we attempted to homogenize the groups as much as possible and in order to increase the precision of the study, two control groups were considered for patients with diabetes types I and II.

In qualitative study of cytological smears, because of xerostomy and dehydratation produced in the oral mucosa of diabetic patients, the ground for increasing the opportunistic infections such as candida and consequently, the inflammation will increase [20]. The results of this study indicate to increase in the inflammation and candida in the oral mucosa of diabetic patients compared to those of the healthy persons in buccal mucosa but the difference was not statistically significant in the tongue area which represents the local performance of inflammation. In terms of increase in the inflammation and candida in diabetic patients compared to the healthy persons, the results of our study is somewhat similar to those of Jajarm et. al’s study [1] who reported increasing the inflammation in the tongue area of diabetic patients.

It is believed that the inflammation results in increasing the nuclear size and decreasing the cytoplasm’s size of the oral mucosa cells But this is true just about the young cells [9]. In diabetic patients, the epithelial cells enter aging process more rapidly because of hormonal defect and/or a disorder in its performance. In total, in this study, the decrease in the size of mucleous and in the size of cytoplasm occurred which somehow confirms this issue. If we assume that the inflammation is effective on the quantitative cytomorphometry changes in the oral mucosa, in order to prevent the effect of inflammation in the present study, cytological smears were prepared from two regions of buccal mucosa and the tongue and to examine the cells, all three epithelial layers obtained from cytobrush were used to prepare the smear.

In relation to the qualitative cytomorphometry characteristics (bilobed or multilobed nucleous, karyorrhexis and vacuolization of the cytoplasm) in diabetic patients and the healthy persons, there are contradictory results in different studies but these symptoms include aging process of the above- mentioned parameters in diabetic patients are more than those of our healthy persons.

The results of our study are similar to those of Alberti et al. [9] and Abdolsamadi et al. [12] in this view. Jajarm et al. [1] reported more percentage of karyorrhexis in the tongue with of diabetes type II compared to the healthy persons which confirm the results of the study in the above-mentioned study. In terms of the ratio of the nuclear to the cytoplasm size in the tongue patients with diabetes type II and control II and patients with diabetes type I and control I, there was not statistically significant difference but this difference was statistically significant in buccal mucosa. It is supposed that the effect of hyperglycemia on the oral mucosa, based on the location of cytological smear preparation, can be different perhaps, the volume of sample is effective on the results.

Altogether, it seem is that the reason for difference between the results of different studies is because of difference in the duration of affliction to diabetes, being controlled or uncontrolled of diabetes, control degree of diabetes (good, moderate, weak), the age and the number of participants, time of diabetes diagnosis, the kind of cytomorphometry measurement software and the microscope magnification applied in order to examine the smears and the absence of a specialized method to examine the cytomorphometry smears.

The limitations of the present study include lowering the volume of sample in diabetes type I which is due to small incidence of this disease. It is hoped to perform more complete and comprehensive studies in the future by increase in the volume of sample.

## Conclusion

Diabetes results in creating quantitative and qualitative cytomorphometry changes in the oral mucosa. The type of diabetes (I and II) and its path ophysiology don’t seem effective on these changes.
